# Feasibility Study and Design of a Wearable System-on-a-Chip Pulse Radar for Contactless Cardiopulmonary Monitoring

**DOI:** 10.1155/2008/328597

**Published:** 2008-03-17

**Authors:** Domenico Zito, Domenico Pepe, Bruno Neri, Fabio Zito, Danilo De Rossi, Antonio Lanatà

**Affiliations:** ^1^Radio-frequency and Microwave Integrated Circuits Laboratory (RFLab), Department of Information Engineering (DIIEIT), University of Pisa, Via Caruso 16, 56122 Pisa, Italy; ^2^Dipartimento di Informatica, Matematica, Elettronica e Trasporti (DIMET), Università “Mediterranea” di Reggio Calabria, Via Graziella, Feo di Vito, 89060 Reggio Calabria, Italy; ^3^Interdepartmental Research, Center E. Piaggio, University of Pisa, Via Diotisalvi 2, 56122 Pisa, Italy

## Abstract

A new system-on-a-chip radar sensor for next-generation wearable wireless interface applied to the human health care and safeguard is presented. The system overview is provided and the feasibility study of the radar sensor is presented. In detail, the overall system consists of a radar sensor for detecting the heart and breath rates and a low-power IEEE 802.15.4 ZigBee radio interface, which provides a wireless data link with remote data acquisition and control units. In particular, the pulse radar exploits 3.1–10.6 GHz ultra-wideband signals which allow a significant reduction of the transceiver complexity and then of its power consumption. The operating principle of the radar for the cardiopulmonary monitoring is highlighted and the results of the system analysis are reported. Moreover, the results obtained from the building-blocks design, the channel measurement, and the ultra-wideband antenna realization are reported.

## 1. INTRODUCTION

In February 2002, the Federal Communications Commission (FCC) gave the permission for the marketing and operation of a new class of products incorporating ultra-wideband (UWB) technology [1]. The FCC, through a modification of the 47 CRF Part 15 regulations [2], decided to allocate for the UWB systems an unlicensed band 7.5 GHz wide (for the first time, in a nonexclusive way), in the range of the radio-frequency spectrum 3.1–10.6 GHz. Moreover, the FCC allocates the unlicensed radio-frequency spectrum between 22 and 29 GHz, for UWB short-range vehicular radars.

Since UWB systems have been released to operate in regions of spectrum in which other services are already operating, the mask of the maximum power spectral density (PSD) allowed for UWB devices has been set to very low values (−41.3 dBm/MHz in the 3.1–10.6 GHz band).

UWB devices can be employed for several applications: ground penetrating radar (GPR), medical imaging, wall imaging, through-wall imaging, surveillance, and high data rate communication systems.

One of the most promising class of applications of the UWB systems consists of the medical imaging (field disturbance sensors designed to detect the location or the movement of objects within the body of a person or animal [2]). In particular, a UWB radar sensor can be employed to monitor the heart wall and chest movements, in order to detect in real time the heart and breath rates, respectively.

The idea at the base of the work presented herein, which has been presented recently [3], consists of the realization of a novel wearable wireless interface for the monitoring of the heart beat and breath rates. This work is a part of the ProeTEX Project (FP6-2004-IST-4-026987), a European integrated project aimed at developing a new generation of equipments for the market of emergency operators, like fire-fighters and civil protection rescuers.

Modern silicon technologies (e.g., transistors of the standard CMOS 90 nm technology of ST Microelectronics have cutoff frequencies higher than 150 GHz) allow us to realize miniaturized and ultra-small- and ultra-low-power wireless UWB sensors for WBAN (wireless body area network) applications. WBANs consist of sensor networks, in which a set of small sensors is placed around the human body or implanted in it, in order to monitor constantly the vital parameters and movements of the person under observation. The information collected by these sensors can be sent, by means of radio-frequency data link, toward remote data acquisition and signal processing units or even to a personal server, which can forward the data to the medical centers and hospitals by means of the Internet. In this way, the medical staff can investigate the manifesting of the heart diseases over all the daily activities of the subject under observation.

With respect to the preliminary study reported in [3], several details concerning the system analysis have been dealt with and reported in this paper. Moreover, this paper reports the advances and the present status of this research. In particular, the performance of the building blocks designed on silicon and the experimental characterization of the intrabody channel and the UWB antenna are reported. The paper is organized as follows. In Section 2, a system overview of the next-generation wearable wireless interface for the heart monitoring is presented. In Section 3, after a brief introduction on the techniques adopted to monitor the heart activity, the principle of operation of UWB radar sensor for the heart monitoring is highlighted. In Section 4, the feasibility study of the UWB radar sensor is discussed. In Section 5, a survey on the channel-loss measurements and the building blocks of the radar is reported, and future developments are discussed. Finally, in Section 6, the conclusions derived by this work are drawn.

## 2. WEARABLE WIRELESS INTERFACE FOR HEART MONITORING: SYSTEM OVERVIEW

The aim of the presented work consists of realizing a novel wearable wireless interface for the heart monitor.

**Figure 1:**
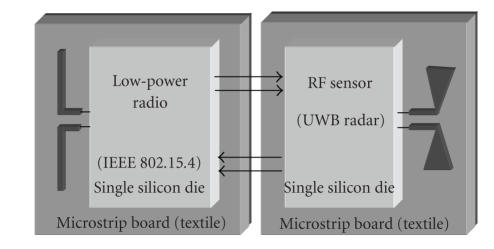
Wearable wireless interface for the heart monitoring: system idea.

The overall system idea is shown in Figure 1. It consists of a fully integrated UWB radar sensor and a low-power radio interface, where each section is realized on a single silicon die. The radar sensor and the low-data-rate wireless transceiver are implemented in a standard CMOS 90 nm technology by ST Microelectronics.

**Figure 2:**
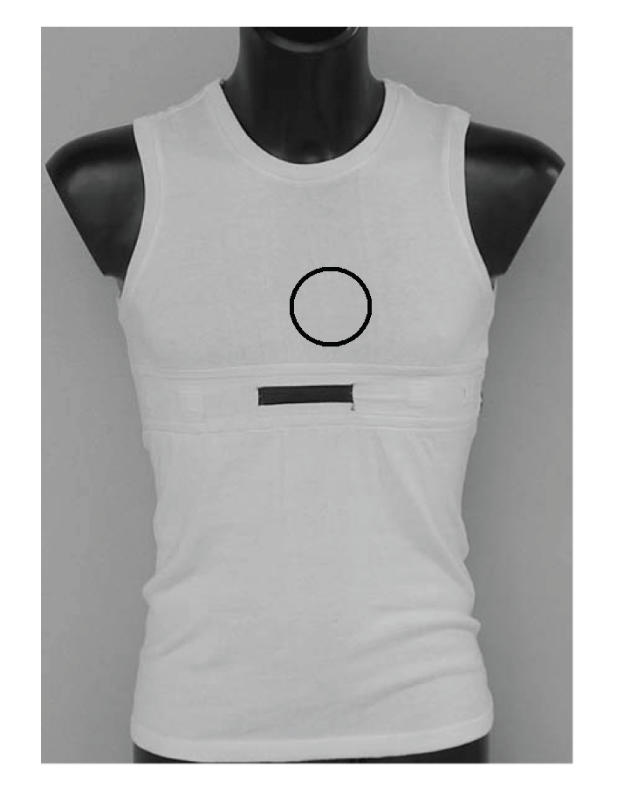
Prototype of the inner garment in which the wearable wireless interface for the detection of the heart and breath rates will be included. In detail, the sensor will be placed around the circled area.

By referring to the scheme of Figure 1, each antenna is realized on a microstrip substrate; however, in the most advanced realization, they can be realized directly by means of proper conductive layer tissues within clothes [4]. These interfaces will be inserted into an inner garment worn by emergency operators, which is shown in Figure 2.

The data acquired by the UWB radar sensor are transferred to a personal or remote unit by means of the low-power radio data link realized by a wireless transceiver based on the IEEE 802.15.4 (ZigBee) standard. By means of the low-power transceiver, the UWB radar sensor can be remotely programmed, increasing the flexibility of the system.

**Figure 3:**
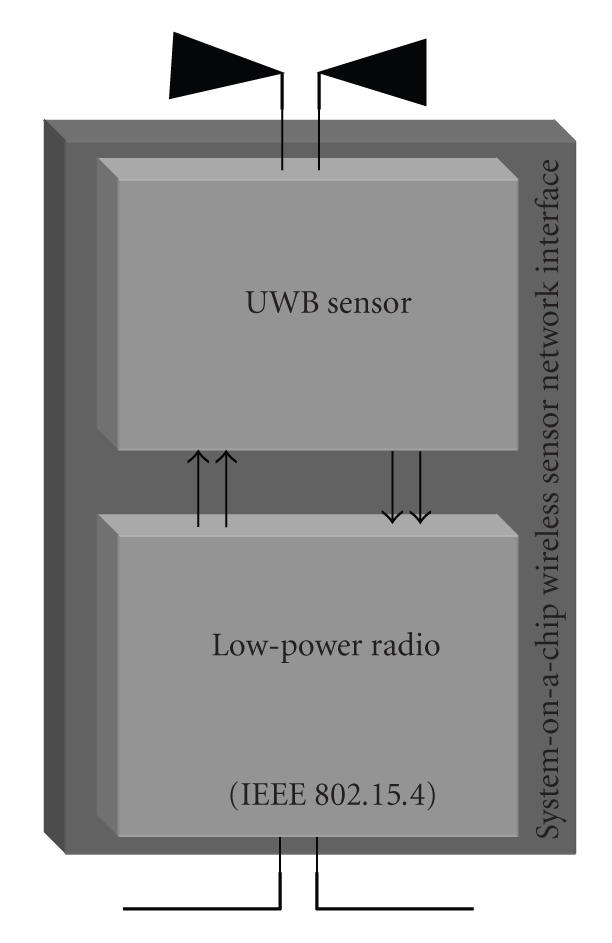
Future perspective: integration on a single silicon chip of the overall wearable wireless interface on a single silicon chip.

A future perspective is the realization of both the radar sensor and the low-power transceiver on the same silicon die (see Figure 3), in order to raise the level of miniaturization and to reduce further on the final costs of the system.

## 3. UWB RADAR SENSOR: PRINCIPLE OF OPERATION

A survey on the current techniques for the cardiac monitoring and the novel radar system proposed in this paper are reported hereinafter.

### 3.1. Radar sensors for monitoring the cardiac activity

The most widespread system for the monitoring of the cardiac activity is the electrocardiograph (ECG). The information provided by ECG is related to heart electrical activity. Pulse oximetry allows us to detect the cardiorespiratory activity, by measuring the saturation level of the oxygen in the blood. Other systems for the monitoring of the cardiac activity are based on ultrasounds (echocardiograph or echo Doppler). Ultrasound-based systems are generally cumbersome and they can be used only by specialized operators. Anyway, all the presented measurement techniques require the direct contact with the body in order to carry out the measurement.

Unlike the traditional techniques (electrocardiograph, ecocardiograph, and pulsed oximetry), radar systems allow the monitoring of the heart activity in a noninvasive and contactless way for the patient [5]. Microwave Doppler radars have been used to detect the respiration rate since 1975 [6]. These first devices were bulky and expensive, but in recent times CMOS fully integrated versions of a radar for noncontact cardiopulmonary monitoring have been presented [7].

Doppler radars typically transmit a continuous wave signal and receive the echo reflected by the target. The frequency of the reflected signal varies from that of the transmitted one by an amount proportional to the relative velocity of the target with respect to the radar.

Another class of radar employed for the monitoring of vital parameters is based on pulse transmission. Pulse radars operate by sending short electromagnetic pulses and by receiving the echoes reflected by the target. The time delay between the transmission of the pulse and the reception of the echo is proportional to the distance from the target to the radar. Discrete prototypes of pulse radar for the detection of vital parameters are reported in literature [8, 9].

It is worth mentioning that radar sensors monitor the mechanical movement of the heart wall instead of the electrical activity of the heart (as the electrocardiograph), and then diseases of the heart that does not show anomalies in the electrical activity can be discovered as well. Moreover, the UWB pulses are not influenced by blankets or clothes [8].

From a circuit design point of view, UWB transceivers present a lower complexity with respect to traditional radio-frequency system, leading to a low-power consumption for a long life of the battery. In fact, with respect to the latter, UWB systems do not require a stable frequency reference, which typically requires a large area on silicon die and consumes a high amount of power.

Moreover, the extremely low level of transmitted power density (lower than −41.3 dBm/MHz) of the UWB radar should reduce the risk of molecular ionization [10–15].

### 3.2. UWB (3.1–10.6 GHz) radar sensor for medical applications: principle of operation

**Figure 4:**
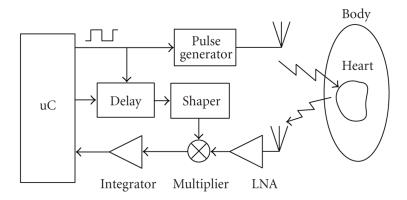
Block diagram of the proposed fully integrated UWB radar for the detection of heart and breath rates.

The main block of the novel wearable wireless interface for human health care described herein is the UWB radar sensor. The block diagram of the proposed radar sensor for the detection of the heart and breath rates is shown in Figure 4. The radar exploits a correlation-based receiver topology followed by an integrator, which averages the received pulses in order to have an output signal containing the information on the heart and breath tones.

The operating principle of a cross-correlator radar is explained hereinafter. An electromagnetic pulse is transmitted toward the target. The echo received from the target is multiplied with a delayed replica of the pulse transmitted; the output signal of the multiplier is then integrated. Note that the output signal will reach its maximum in the case of perfect time alignment between the two signals at the input of the multiplier itself. In other terms, the cross-correlator has a frequency response equal to that of a matched filter. In particular, it can be demonstrated that the matched filter is the filter that allows to obtain the best signal-to-noise ratio at the output [16]. Moreover, this has been confirmed by preliminary system simulations (by means of the Ptolemy simulator within Agilent ADS2005A). In detail, the CAD system analysis has shown that this topology allows us to achieve the best performance in terms of output signal-to-noise ratio (SNR) and sensitivity to small variations of the position of the heart wall with respect to other topologies, like that in which the receiver is simply turned on by the command given by the delayed replica of the transmitted pulse [8, 9].

The principle of operation of the overall radar system shown in Figure 4 is explained hereinafter. A train of extremely short (about 200 picoseconds) Gaussian monocycle electromagnetic pulses is transmitted toward the heart. Since the heart muscle and the blood that flows inside have different characteristic impedance, a partial reflection of the energy associated with the radiated pulse occurs at the surface of separation of these two different media [8].

After a time delay approximately equal to the flight time of the pulse from the transmitter to the receiver (about two nanoseconds), a delayed replica of the transmitted pulse generated internally (by the delay and shaper blocks) is multiplied with the echo received. It is worth mentioning that, in practice, the shaper block can be replaced by an additional pulse generator. A pulse repetition frequency (PRF) greater than 1 MHz allows us to consider the heart almost “motionless” between two consecutive pulses. The amplitude of the signal at the output of the multiplier reaches its maximum when the received echo and the delayed replica of the transmitted pulse are perfectly time aligned. If the time delay is fixed, a fixed range gate is monitored by the radar, and the amplitude of the signal at the output of the multiplier is related to the position of the heart. The output voltage of the receiver front end is averaged by integrating over a large number of pulses. This operation allows us to increase considerably the signal-to-noise ratio at the output of the receiver, as explained in Section 4.2. Moreover, the amplitude of the continuous signal at the output of the integrator is related to the time-varying position of the moving object under observation, that is the heart wall in our case. Therefore, the output signal provided by the integrator includes the tones of the heart beat and breath frequencies.

## 4. FEASIBILITY STUDY OF THE UWB (3.1–10.6 GHZ) RADAR SENSOR

A theoretical model of the intra-body channel in which the electromagnetic pulse propagates has been developed and a complete feasibility study of the UWB radar sensor has been carried out in order to demonstrate the effectiveness of such an approach. The specifications of the single blocks of the radar have been derived by taking into account the performance achievable by means of its implementation in a standard CMOS 90 nm technology by ST Microelectronics. System simulations have been performed by means of CAD tools in order to verify the feasibility of the proposed UWB sensor radar. In particular, CAD tool system analysis has been carried out by including accurate models at different levels of abstraction and nonideal effects (noise contribution and bandwidth limitation of the building blocks) associated with the technology process.

### 4.1. A simple theoretical model of the channel

A simple theoretical model of the channel has been developed in order to derive the system specifications of the fully integrated radar sensor, and then simulate the overall radar system.

A frequency-dependent channel-loss model (in the band 1–12 GHz) has been developed. The overall loss (*L*(*f* )) has been calculated by taking into consideration the contributions of the following: (i) path loss (PL(*f* )); (ii) attenuation in the tissues (Att(*f* )); and (iii) losses due to the reflections at the interface between different tissues (Rfl(*f* )). The properties of the body tissues have been extracted by the parametric model of the dielectric properties of the body tissues developed by C. Gabriel et al. at Brooks Air Force Base (USA) [17, 18]. As for the thickness of the tissues layers, the model proposed in [5], based on the Visible Human Project and Gabriel's data book, has been considered. An antenna gain equal to 1.8 (2.5 dB) has been chosen to determine the contribution of the path loss, by referring to a UWB antenna realized, which has been proposed in [19]. Since we rely on applying the system-on-a-chip radar, and thus its antenna, very close to the skin in proximity of the chest, near field equations [20, 21] have been used in order to evaluate the path and reflection losses.

**Figure 5:**
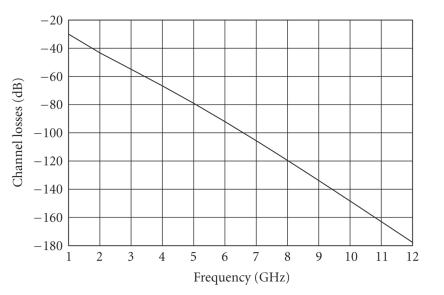
Channel loss versus frequency predicted by the near-field-based model.

The detailed formulas employed to derive the frequency-dependent channel-loss model have been reported in [22]. The result is summarized herein (see Figure 5).

Simulation results show that the average power loss of the pulse in the 3.1–0.6 GHz band amounts to about 80 dB. Note that this result is in agreement with the channel measurements reported in [23].

The time of flight of the pulse has been estimated in about two nanoseconds (in accordance with the data reported in the literature [8]). The maximum time difference in the flight time of the pulses due to the heart displacement (1-2 cm) has been estimated in a few hundreds of picoseconds.

To validate this channel-loss model, a set of several antennas each operating in a different frequency subrange has been realized in order to perform channel-loss measurements over the frequency range of interest. The results are described in Section 5.

### 4.2. Theoretical system analysis and specifications

As for the transmitted electromagnetic pulse, it has to be very short in order to have a range resolution in the order of the centimeter, since the maximum displacement of the heart wall is of a few centimetres (1-2 cm). A duration time (*τ*) of the pulse of about 200 picoseconds makes possible the achievement of an accurate range resolution. Thus the maximum of the power spectral density results would be at 5 GHz (within the 3.1–10.6 GHz band), since the maximum of the spectrum of a Ggaussian monocycle pulse is placed at the frequency 1/*τ*.

The Gaussian monocycle has been preferred to the Gaussian pulse because it has no dc component and its spectrum matches the FCC emission level mask. Furthermore, the Gaussian monocycle pulse can be implemented efficiently on silicon by means of differential transmitter [24]. The peak-to-peak voltage of the pulse has been chosen equal to 1.2 V, by considering that the radar will be realized in a standard CMOS 90 nm technology by ST Microelectronics, which is characterized by a power supply of 1.2 V (this is not a critical value since the theoretical maximum peak-to-peak voltage of the pulse generated by the differential transmitter amounts to the double of the supply voltage, without considering the voltage drop in the transistors).

As for the receiver, if SNR_out_ is the advisable output signal-to-noise ratio, the minimum power (*S*_min_) required at the input of the receiver amounts to

(1) $$S_{\min}=k\times T\times B\times \text{NF}\times \text{SN}{\text{R}}_{\text{out}},$$

where *k* is the Boltzmann constant (1.38 × 10^−23^), *T* is the equivalent temperature of the antenna, NF is the receiver noise figure, *B* the bandwidth of the receiver, and SNR_out_ is the signal-to-noise ratio at the output of the receiver. If we consider an average channel loss equal to 80 dB in the 3.1–10.6 GHz band, a noise figure lower than −11.5 dB would be required in order to have an output SNR greater than 10 dB (for a single pulse).

This result is clearly not reachable in practice, so that an improvement is required. The output SNR of the radar receiver can be increased by integrating several pulses. The characteristic frequencies of the vital parameters under observation are within a few Hertz, then an integrator band of 100 Hz is wide enough to keep appropriately the heart and breath rates. The case of an integrator with a bandwidth of 1 KHz and a PRF in the range 1–10 MHz has been investigated. For a PRF equal to 10 MHz, 10 000 pulses are averaged. In particular, an integration over 10 000 pulses allows an improvement of the SNR (SNR_imp_) at the output of the receiver of about 40 dB, as shown in the tables reported in [25]. This result is confirmed by the rough estimation given by the following equation:

(2) $$\text{SN}{\text{R}}_{\text{imp}}\approx 10\times \log\left(\frac{\text{PRF}}{B_{\text{int}}}\right)=10\times \log10 000=40\;\text{dB,}$$

where *B*_int_ is the bandwidth of the integrator. The receiver front-end (LNA and multiplier) specification results are to be thus relaxed by the integration of a great number of pulses. In these conditions, the noise figure of the receiver front end has to be lower than

(3) $$\text{N}{\text{F}}_{\max}=-11.5+40=28.5\;\text{dB}\text{.}$$

The specifications for the LNA are set in a power gain of 15 dB and a noise figure of 5 dB, whereas for the multiplier, a voltage gain of 0 dB and a noise figure of 10 dB. With these specifications, the noise figure of the overall receiver front end will result in largely lower than the maximum NF allowed for a proper detection. It is worth mentioning that the aforementioned specifications can be obtained by an implementation in a standard CMOS 90 nm technology.

Within this framework, a large integrator voltage gain is required. In fact, the dc component of the signal at the output of the multiplier, in the case of perfect time alignment between the signal received and the replica delayed of the transmitted pulse, has been estimated in a few hundreds of nanovolts. Thus, an integrator gain equal to 120 dB is required in order to have an amplitude voltage of a few hundreds of millivolts at the integrator output.

### 4.3. System analysis by means of CAD tool simulations

The overall radar system has been simulated by means of the Ptolemy simulator within the CAD tool ADS2005A by Agilent Technologies. Each block of the overall radar system has been implemented in the simulator by functional blocks which take into account their bandwidth limitations and noise contributions.

The channel model (of the human chest) has been implemented as a frequency-dependent *S*-parameter block, which has been included in the system analysis of the overall radar. This parameter description provides an equivalent behavior in terms of frequency response of the theoretical channel model shown in Section 4.1.

The simulation results show that the transmitted pulse is strongly distorted by the channel. To be noted that in narrow-band radar, the reflected wave has almost that same shape of the transmitted: it keeps the sinusoidal shape for signal down-conversion such as addition, subtraction, differentiation, and integration. In the case of UWB radar, the signal changes its shape during transmission, channel propagation, and reception and this impairs the signal-to-noise ratio.

An additional block realizing a periodically variable flight time has been included in order to emulate the cardiac movement. For the sake of clarity, this block emulates only two positions of the heart, providing a difference of 200 picoseconds in the arrival time of the pulse received for the two positions. The heartbeat period has been set in 20 milliseconds (this has been reduced with respect to the real heart movement in order to reduce the simulation time). However, this is not a limitation since the radar system reaches the steady state within ten milliseconds, thus the simulation results in terms of output signal and output SNR will not be influenced by this assumption.

Moreover, an additional white thermal noise source has been included in the channel in order to take into account the antenna noise at the input of the receiver. Both antennas, that of the transmitter and that of the receiver, have been included in the simulator by a 7.5 GHz band filter (centred at 6.85 GHz, with a slope of −20 dB/dec) each. The LNA block has a band of 3.1–10.6 GHz and an out-band slope of the frequency response equal to −20 dB/dec.

A two-step simulation has been carried out, since the time constants of the radio-frequency and the baseband parts of the radar are quite different. Actually, the radio-frequency part has to be simulated with a timestep of about one tenth of the pulse width (i.e., the duration time), whereas the baseband part requires a simulation time of at least several tens of milliseconds. A timestep of 10 picoseconds has been adopted for the RF section. The integrator (with 120 dB of gain and 1 KHz of band) has been splitted in three low-pass filters with gain equal to 40 dB and band equal to 10 MHz, 100 KHz, and 1 KHz, respectively.

Simulation results are in agreement with those obtained by theoretical system analysis. In particular, the simulations show that the increase of the SNR from the output of the multiplier to the output of the integrator is of about 40 dB, as predicted by the theoretical system analysis.

**Figure 6:**
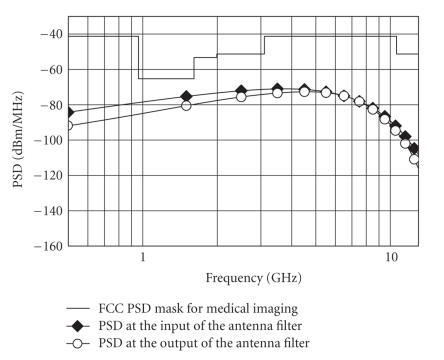
Power spectral density (PSD) of a pulse sequence with PRF equal to 1 MHz versus frequency.

**Figure 7:**
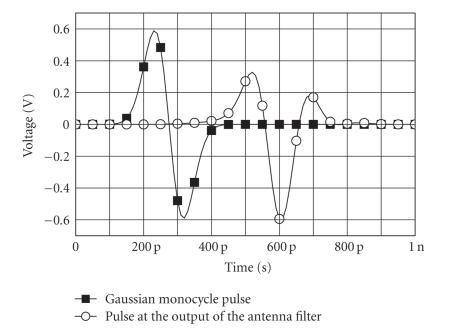
Gaussian monocycle pulse and pulse at the output of the antenna.

**Figure 8:**
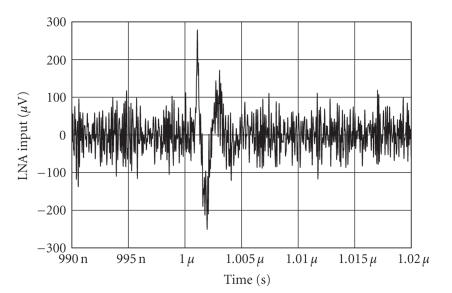
Voltage signal at the input of the LNA, including the noise contributions.

**Figure 9:**
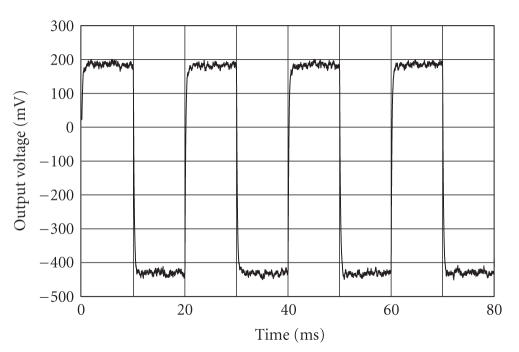
Voltage at the output of the integrator. The output signal has the same frequency of the movement imposed for the heart wall. A time-varying surface with a period of 20 milliseconds has been considered for the simulation (this period is short with respect to the real heart moving, in order to reduce the simulation time. This does not impair the analysis since the radar reaches widely the steady state within ten milliseconds).

Figure 6 shows the following: (i) the power spectral density (PSD) of the pulse sequence with duration time of 200 picoseconds, 1.2 Volt peak-to-peak amplitude, and PRF equal to 1 MHz, at the input of the antenna filter; (ii) the power spectral density (PSD) of the pulse sequence resulting and PRF equal to 1 MHz, at the output of the antenna filter; and (iii) the FCC mask for the UWB medical imaging applications. The Gaussian monocycle pulse at the input of the transmitter antenna filter and the pulse obtained at the output of the antenna filter are shown in Figure 7. The pulse at the input of the receiver (i.e., the output of the antenna filter of the receiver) is shown in Figure 8. Note the large amount of noise superimposed to the advisable signal. The output voltage of the integrator is shown in Figure 9. Note that the output signal reaches two different voltages for the two different positions of the heart.

It is worth mentioning that similar performance has been obtained by using sinusoidal pulses with the same duration and amplitude.

## 5. PRESENT STATUS AND FUTURE WORKS

Present and future works are addressed to the building blocks design in CMOS 90 nm technology by ST Microelectronics and their cointegration, UWB antenna design, channel model verification by means of measurements, system-on-a-chip prototyping, and experimental characterization.

A set of antennas resonating at the frequencies of interest has been realized in order to perform channel measurements. The results of these measurements are hereinafter reported.

Moreover, preliminary prototypes of UWB antenna have been realized.

The most critical radio-frequency blocks of the radar (low noise amplifier, pulse generator, and multiplier) have been designed and sent to the silicon foundry for the test chip prototyping.

A summary of the most relevant results achieved at the present status is reported.

### 5.1. Channel-loss measurements

Experimental characterizations of the intrabody channel have been reported in the literature [23]. It is important to remark that results obtained by our simple channel model have shown a wide agreement with those measured in [23]. In detail, this work reports that the mean loss in the UWB band between two antennas placed towards the chest on the same side is of about 80 dB.

**Figure 10:**
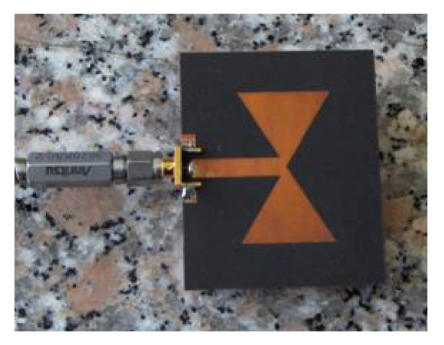
Antenna prototype for the channel model verification.

In order to verify definitively through measurements the theoretical channel model we have developed, and to investigate some details concerning our application (e.g., the distance antenna-skin for a proper antenna operation), a set of antennas resonating at different frequencies in the spectrum range of interest has been realized (an example is shown in Figure 10). The antennas have been designed by means of Momentum, the EM simulator within Advanced Design System (ADS) by Agilent Technologies.

**Figure 11:**
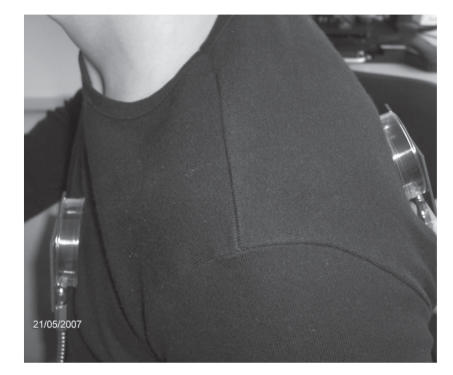
Setup for the channel-loss measurement, setup between the front and the back of a human chest.

The measurements have been carried out using a vector network analyzer (VNA) placing two identical antennas, one in front of the chest of the subject under test (SUT) and the other on the back of the chest Although this measurement setup is not directly related with the antenna-heart-antenna path we modelled, this allows us to extract information concerning the attenuation in the tissues, avoiding any systematic error caused by proximity effects (i.e., direct coupling) of the two antennas placed on the same side. Each antenna irradiates towards the other antenna. The measurement setup is shown in Figure 11. The antennas are placed at one cm of distance from the skin (by means of plastic structure, which does not impair the electromagnetic behavior). Several tests have been carried out by varying the distance between the antenna and the skin surface, showing that the measurement results obtained do not significantly vary for distances of more than about five millimeters. If the distance antenna-skin decreases, then the radiation pattern of the antenna changes and the attenuation increases. This result is in agreement with the measurement reported by other researchers in [4].

**Figure 12:**
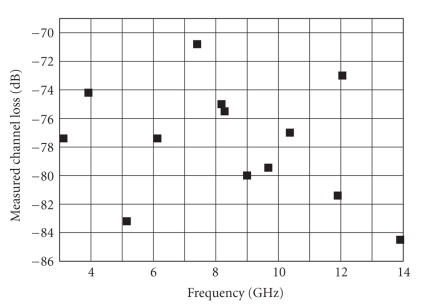
Measurement results of the intrabody channel loss.

The results of our measurements (and the resonating frequencies of the set of antennas realized) are reported in Figure 12. The mean value of |S21| has resulted approximately equal to 78 dB. Note that the antennas we designed have a gain of about ten dB. Thus if we consider the typical path loss of a UWB antenna characterized by a gain of 2.5 dB (as considered in our channel model [22]), an additional loss of about 15 dB (10–2.5 dB for each antenna) has to be summed up. This means that the overall loss measured amounts approximately to 93 dB.

A direct comparison with the result (109 dB) reported in [23] cannot be carried out since, therein, the measurements have been carried out by means of antennas at contact with the skin. In fact, this causes an additional loss due to the change of the radiation pattern of the antenna itself (see above). Moreover, in [23], the directivity of the antennas used to carry out the test is not reported. Regardless of that, the two results are reasonably close to each other.

Anyway, the experimental results, in spite of being derived from a different setup, are in the order of those predicted by the model, especially in the lowest part of the 3.1–10.6 GHz frequency band. The increase of the attenuation at the upper part of the frequency spectrum predicted by the model is mitigated (in the measured results) by the increase of the antenna efficiency of the antennas realized.

Finally, this experimental investigation shows that the UWB radar, and especially its antenna, can be realized as wearable device close to the human body.

### 5.2. UWB antenna

**Figure 13:**
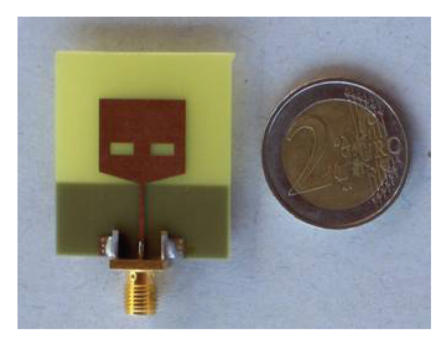
Prototype of UWB antenna realized.

Preliminary UWB antenna prototypes have been realized. Knight's helm shape antennas [26] have been realized in order to implement the wideband antenna. One of the antenna prototypes is shown in Figure 13.

The antenna has been realized on a microstrip substrate. As for the antenna, it is worth mentioning that the feasibility of textile UWB antennas has been demonstrated in [4].

**Figure 14:**
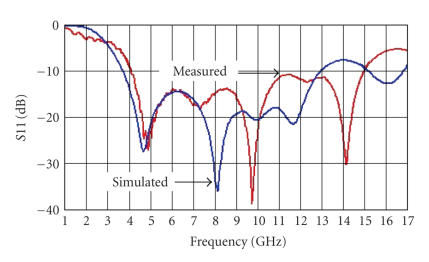
Simulated (blue) and measured (red) S11 parameters of the UWB antenna of Figure 13.

The simulated and measured parameter S11 of this antenna is shown in Figure 14. It can be seen that the −10 dB band is between 4.081 GHz and 14.95 GHz. Note the large agreement between measurements and simulations up to the 7.5 GHz. However, the mismatch in the higher region of frequency (from 7.5 to 10.6 GHz) occurs and it is due to the approximations (2D and 1/2) of the geometry introduced by the EM simulator. The fractional bandwidth (*B*%), can be defined as follows:

(4) $$\begin{array}{cc} B\% & =\frac{B}{f_{c}}\times 100,\\ f_{c} & =\frac{f_{\max}+f_{\min}}{2}. \end{array}$$

Then note that the fractional bandwidth results are equal to 114.22%.

Present and future works on this task are addressed to realize a UWB antenna on a textile substrate with performance perfectly matched with the UWB band.

### 5.3. Building blocks in CMOS 90 nm by ST Microelectronics

A UWB 3.1–10.6 GHz low-noise amplifier (LNA) in CMOS 90 nm process by ST Microelectronics has been designed. The LNA consists of a common gate input stage and two subsequent common source gain stages. The common gate input stage allows the realization of a wideband input integrated matching to the source impedance of the antenna. This novel topology allows the achievement of a wideband input integrated matching by overcoming the issues of the classical wideband input matching technique implemented with passive low-pass filters, such as the parasitic effects and noise contributions introduced by the passive elements at the LNA input [27].

Schematic is reported therein [27]. With respect to the solution presented in [27], a three-stage topology has been adopted in order to reach the specifications in terms of gain.

If compared with other solutions with the same power consumption, the proposed LNA exhibits a very good performance tradeoff between gain, bandwidth, noise, and linearity. The design does not present any critical issues and exhibits an excellent design reliability.

Postlayout simulations have shown that the presented LNA provides a transducer gain (GT) of 19.34 dB at 5.4 GHz and a −3 dB band from 3.4 GHz to 8.3 GHz. Within the UWB band, the S11 parameter is equal to −22.33 dB at 3.1 GHz and −4.09 dB at 10.6 GHz. The noise figure (NF) is equal to 6.24 dB at 3.1 GHz and 5.13 dB at 10.6 GHz.

The overall LNA draws 41.71 mA on a 1.2 V power supply (biasing network included).

A novel fully integrated UWB pulse generator has been also designed in CMOS 90 nm process by ST Microelectronics [28]. Schematic is therein [22]. The pulse generator provides monocycle pulses with duration time close to 250 picoseconds and 1-V peak-to-peak amplitude. In detail, the circuit provides a sinusoidal-like monocycle when activated by a negative edge of a trigger signal provided by a microcontroller. This activation can be delayed in the range 1–3 nanoseconds, by acting on a 5-bit programmable delay element, which provides a total set of 32 different delay times.

This new pulse generator provides a sinusoidal-like monocycle pulse with 1-V peak-to-peak and 250 picoseconds of duration time on a differential antenna with input impedance equal to 100 Ohm.

The overall pulse generator consumes 22 mW on a 1.2 V power supply, which represents an extremely low-power dissipation with respect to the other solutions presented in the literature.

The multiplier consists of a PMOS input matching stage, followed by a PMOS double balanced Gilbert cell terminated on a capacitive load, representing the input impedance of the following gain stage. Simulation results show a conversion gain G_C_ equal to 1.71.

To be noted that the performance of all the building blocks is in line or widely within the system requirements. For the sake of clarity, it has to be mentioned that the specifications derived for the receiver were more stringent than those strictly required for a proper operation.

Present work on this task is addressed to the design of the delay generator and integrator (note that feasibility has been widely demonstrated in several works presented in literature [29, 30]), and the cointegration of all the building blocks into a single silicon die.

## 6. CONCLUSIONS

The recent advances in silicon CMOS technology allow the realization of more and more miniaturized, low-cost, and low-power integrated system-on-a-chip sensors. These sensors can be employed in the wireless body area networks, for advanced and continuous monitoring of vital parameters.

In particular, the system overview of next-generation wearable wireless sensors for human health care and safeguard has been presented herein. Such a system is composed by a novel fully integrated ultra-wideband radar sensor for the detection of the heart and breath rates and a low-power radio interface (IEEE 802.15.4, ZigBee), which collects the data provided by the sensor and sends these data to a remote data acquisition unit or even in the Internet by means of a personal server. Thus the physiological data of a person under observation can be sent in real time to the hospital to be analyzed and then the doctors could act in time in case of anomalies in the vital parameters monitored.

A detailed feasibility study of the UWB radar on silicon technology (CMOS 90 nm) has been carried out, by means of both theoretical analysis and CAD tool simulations. The simulation results have shown a wide agreement with the theoretical model of the radar, demonstrating the feasibility of the proposed system-on-a-chip radar in a modern silicon technology.

Moreover, the main critical building blocks of the radar sensor have been designed and prototyped in CMOS 90 nm process by ST Microelectronics. Channel-loss model has been confirmed by means of several tests in laboratory. In particular, measurement results of the intrabody loss have shown results in quite agreement with the predicted ones especially in the lower portion of the 3.1–10.6 GHz band. Preliminary prototypes of UWB antennas have been realized and characterized to be implemented on a textile substrate.

Present and future works are addressed to the design of the noncritical building blocks and final system-on-a-chip prototyping, the cointegration with the textile antenna, and the medical characterization in clinical environment of this innovative UWB microsensor for contactless and noninvasive cardiopulmonary monitoring.
